# ‘Generic visuals’ of Covid-19 in the news: Invoking banal belonging through symbolic reiteration

**DOI:** 10.1177/13678779211061415

**Published:** 2022-07

**Authors:** Giorgia Aiello, Helen Kennedy, C.W. Anderson, Camilla Mørk Røstvik

**Affiliations:** School of Media and Communication, 4468 University of Leeds, UK; Department of Philosophy and Communication, 9296 University of Bologna, Italy; Department of Sociological Studies, 7315 University of Sheffield, UK; School of Media and Communication, 4468 University of Leeds, UK; School of Divinity, History, Philosophy & Art History, 1019 University of Aberdeen, UK

**Keywords:** banal nationalism, Covid-19, data visualization, generic visuals, news media, symbolic reiteration, stock photography, visual communication

## Abstract

In the early days of the Covid-19 pandemic, images of the virus molecule and ‘flatten-the-curve’ line charts were inescapable. There is now a vast visual repertoire of vaccines, people wearing face masks in everyday settings, choropleth maps and both bar and line charts. These ‘generic visuals’ circulate widely in the news media and, however unremarkable, play an important role in representing the crisis in particular ways. We argue that these generic visuals promote banal nationalism, localism and cosmopolitanism in the face of the crisis, and that they do so through the symbolic reiteration of a range of visual resources across news stories. Through an analysis of three major news outlets in the UK, we examine how generic visuals of Covid-19 contribute to these banal visions and versions of belonging and, in doing so, also to foregrounding the role of the state in responding to the crisis.

## Introduction

In the early days of the Covid-19 pandemic, images of the virus molecule and ‘flatten-the-curve’ line charts were inescapable. Since early 2020, this visual landscape has changed creating a much broader iconography of the continued crisis. There is now a vast visual repertoire of vaccines, people wearing face masks in a range of everyday settings, choropleth maps and bar charts – first of case rates and then of vaccination rates. These ‘generic visuals’, namely stock photos and simple data visualizations, circulate with increasing frequency in the news media and, however mundane and unremarkable, play an important role in representing and mediating the Covid-19 pandemic in particular ways. During a time of global crisis like that which we have experienced in the past two years,we suggest that a critical investigation of generic visuals enables understanding of some of the emergent ideologies of Covid-19, specifically with regard to the relationship between political power and public interest.

This article advances four claims about the role of generic visuals of Covid-19. First, we argue that we ought to broaden and deepen our understanding of generic visuals, exploring the semiotic and communicative work that they do during events like the Covid-19 crisis. Second, per Michael Billig's concept of ‘banal nationalism’, we argue that different types of generic visuals of Covid-19 promote banal visions and versions of nationalism, localism and cosmopolitanism in the face of the crisis, in different but connected ways. Third, they accomplish this through symbolic reiteration, or the performative repetition and resignification of a range of visual resources across news stories and other sources. Fourth, by invoking the nation, generic visuals of Covid-19 also foreground the role of the state in grappling with the crisis, in ways made possible by their continued symbolic reiteration. In making this point, we also empirically document the growth of state intervention as a result of the pandemic – what Gerbaudo (2021) has called ‘neo-statism’ – and the ways this relates to ideas of banal belonging.

The data we draw upon is based on analysis of news visuals from three major news outlets in the UK, at distinct moments during the pandemic: December 2020/January 2021 and April/May 2021. These were major moments in the Covid-19 crisis, specifically in the UK, as the first was at the height of the second wave against the backdrop of Brexit, while the second coincided with the UK's effective rollout of mass vaccinations. The three outlets analyzed were Reach PLC (formerly the Trinity Mirror Group), chosen because it publishes a range of regional and national tabloid newspapers; the *Financial Times* (or *FT*), chosen because it is a broadsheet publication targeted at a more global and elite audience; and BBC Yorkshire, chosen as a BBC regional office focusing on regional news. We focus on the UK as a lens through which to explore the broader work that generic visuals do.

## Generic visuals in the news: why stock photos and simple data visualizations?

In this article, we take stock photos and simple data visualizations as examples of generic visuals and as starting points for our investigation of visual genericity and the work that it does. We bring these seemingly disparate types of visuals together because we see them both as part of the ambient image environment that defines our visual world (Frosh, 2020), particularly the world of news. Stock imagery and data visualizations are increasingly ordinary in a way that has consequences (Kennedy et al., 2016), and their growing ubiquity vis-à-vis more traditional ‘press images’ highlights the importance of investigating the role that they play in ‘assembling publics’, a term we use, building on Warner (2002), to refer, for example, to bringing groups of people together around shared interests and concerns, activating citizens to care (or not) about particular issues, making possible (or not) various forms of engagement, including democratic decision-making, and in facilitating or inhibiting the spread of disinformation.

News organizations utilize stock photography on a regular basis, often sourcing royalty-free, ready-to-use images from pay-per-image, subscription-based, or even free online image banks (Machin and Polzer, 2015). The uproar generated by a 2018 Poynter Institute tutorial on how to use stock imagery suggests that these forms of visual journalism are simultaneously common, controversial, and little understood (Brown, 2018). Until recently, the use of data visualizations in the news was an elite journalistic practice, epitomized in the award-winning work of *The Guardian* and *The New York Times*. But visualizations are also becoming generic in their visual form, produced with standardized software and conforming to stylistic guidelines. As a result, simple graphic forms like bar charts and line charts are replacing the more experimental earlier visualization work in data journalism. Quantitative information is increasingly turned into standardized visual products, which in turn are productive of particular ways of representing the current events that manifest themselves in the ‘news of the day’ (or the hour, or the minute).

One reason to consider simple data visualizations and stock photographs together as generic visuals is that news organizations *use* them together in rather unique ways. A second justification for combining them in our analyses is that they are both largely absent within scholarly literature on journalism. In the (relatively limited) research that probes the significance of visuals in the news, analyses prioritize arresting or iconic photographs (Zelizer, 2010). Likewise, recent studies of increasingly ubiquitous data visualizations in the news have similarly focused on those that are iconic, award-winning or considered beautiful (e.g. McCosker and Wilken, 2014). One exception to the general tendency of overlooking generic visuals in the news is the collaborative work of David Machin and others, on which we build. Machin and co-authors have emphasized the increasing importance of generic images in journalism's aesthetics across magazine design, print and online newspapers, and television news (Machin and Niblock, 2008; Machin and Polzer, 2015). In doing so, they foreground the key role that generic visuals play in contributing to the overall ‘design’ of news stories – for example, when ‘conceptual’ photos of glass and steel buildings in urban settings are used to signify modernity and business.

Another exception is the work of Paul Frosh. In his book on the visual content industry, Frosh (2003) defined stock photography as the ‘wallpaper’ of media and consumer culture more broadly, highlighting the important role that these pre-produced, ready-to-use images play in limiting much of our everyday visual world to commercially defined stereotypes and clichés. In later work Frosh (2020) also considered the ‘genericity’ of these images as a form of approximation that may not only work to erase the distinctiveness of their subjects but may also serve as a resource for the communication of a range of values and the promotion of pluralistic identities in the media. In other words, generic visuals may contribute to expanding our ways of seeing the world by virtue of their flexibility and lack of specificity (Frosh, 2020). Recent work by one of us and others on stock imagery in news media has highlighted not only their ubiquity (Aiello et. al, 2017), but also how their uses and re-uses have important implications for how we come to ‘see’ particular issues, often in one-sided if not reductionistic ways (Thurlow et al., 2020). 

There has been even less acknowledgement and critical examination of the increasing number of ‘standardized’ data visualizations we encounter in everyday life. One exception of particular importance to our work here is Tal and Wansink's (2016) exploration of ‘trivial graphs’. Tal and Wansink (2016: 124) suggest that ‘[e]ven easily produced, trivial elements’ presented as data visualizations are often believed, persuading by creating ‘a scientific appearance which is not in fact justified by evidence’. This suggests that there is a strong relationship between simple, everyday data visualizations and public trust in science. Elsewhere, two of us have examined the conventions that shape data visualization as a visual communication genre centred on rhetorics of objectivity, transparency, and facticity (Kennedy et al., 2016). A small number of other scholars have also started to document and examine how various types of simple data visualizations – like, for example, bar charts, line charts, choropleth maps, but also, more broadly, diagrams – speak to audiences in different ways (Amit-Danhi and Shifman, 2018; Ledin and Machin, 2018; Weber et al., 2018). As already noted, we are also interested in this question of how audiences are brought into being, assembled as publics (Marres, 2007; Warner, 2002) in our research into generic visuals. It is easy enough to understand how iconic photographs or splashy visuals like the famous *New York Times* piece ‘Snowfall’ might do this work of public assemblage; the role of visual wallpaper in public life is less clear.

On the one hand, there are similarities in the uses of stock imagery and simple data visualizations in the news that ought to be investigated. On the other, there are significant differences between these two different types of visuals, specifically in relation to how they might be perceived by the public. While data visualizations are sometimes seen as serious and trustworthy representations conveying ‘truth’ and ‘facts’ (Kennedy et al., 2016), stock photography has typically been discounted as overly stereotypical, cheap and bland, and it has also often been derided for promoting ridiculous clichés (Aiello, 2016). Therefore, we take simple data visualizations and stock photography as starting points for investigating generic visuals, recognizing that, in bringing them together, we are dealing with two different visual forms and systems of influence which have shared characteristics – that is, they have standardized formats and appearances, perform particular design functions in relation to the layout of online news stories, and circulate with increasing frequency in the news media next to more traditional ‘press images’. As we proceed with our analysis, it may be that bringing them together is productive, or that other image types need to be considered. Regardless, our analysis of different types of generic visuals in the news enables us to foreground the more mundane and overlooked visual dimensions of Covid-19 coverage and of news media more generally.

In the next section of this article, we discuss the visual dimensions of the Covid-19 crisis. We outline a conceptual distinction between iconic and generic visual representations of Covid-19, contrasting the symbolic condensation of the former with the symbolic reiteration of the latter. This conceptual work informs our subsequent analysis of a range of Covid-19 visuals from three major British news media organizations. It also contributes to further refining our definition of generic visuals in the news.

## The visual landscape of Covid-19: the role of generic visuals and their symbolic reiteration

Since the beginning of the Covid-19 global crisis we have been awash in visual representations of the coronavirus, to the extent that we have become accustomed to a daily onslaught of pandemic-themed photo galleries, charts and graphs, in both official and amateur footage across social media and the news. And while the visual landscape of Covid-19 has changed a great deal since the beginning of the crisis, we note elsewhere that there are three examples in particular – the virus molecule, the flatten-the-curve line graph, and the face mask – that have come to represent many issues related to the pandemic, and which continue to circulate widely across a range of visual media (Mørk Røstvik et al., in press for 2022).

As Julia Sonnevend (2020) explains, the now ubiquitous image of the coronavirus molecule is an aesthetically pleasing ‘close-up’ that synthesizes key information about the virus while also being both versatile and relatable, thanks to its economy of detail and tactile quality. Likewise, she adds that the flatten-the-curve line graph has become widespread in part because it is an especially simplified visual rendition of the complex epidemiological relationship between social distancing measures (or lack thereof) and hospitalization rates over time. Alongside the coronavirus image and the flatten-the-curve graph, the face mask has become a ubiquitous visual that stands for the ‘new normal’, that is, the medicalization of everyday life (Leone, 2021). Images of people wearing face masks in a variety of settings – hospitals, airports and schools, but also supermarkets, parks, and city streets – have become the norm in news media, pointing both to the particular moment in history that we are living and the universal nature of the pandemic. Arguably, these three visuals have become iconic of the pandemic. This is because they work as ‘symbolic condensations’ (Alexander, 2008: 782) of what we cannot in fact see with our own eyes – namely the virus, the infection rates, virus transmission. By channelling culturally meaningful issues and historically significant events into aesthetic form, iconic visuals demand attention and invite recollection. In a related manner, these visuals also work as ‘allusions’ to what we are most often not shown: illness and death (Alexander and Smith, 2020). Ultimately, as Sonnevend (2020: 461) states, these are visual representations of the virus which ‘summarize more than they actually depict’.

The virus molecule image, the flatten-the-curve graph, and the face mask visual have been widely adapted to a variety of communicative contexts (Mørk Røstvik et al., in press for 2022), to the extent that we may in fact consider them as much generic as they are iconic. In the arc of just a few months, they have become memorable and immediately recognizable as visuals in their own right, thus becoming pervasive ‘[v]isual commonplaces’ (Hariman and Lucaites, 2007: 2) of the crisis. Ultimately, among the myriad images that have been used in the news media to cover the pandemic since early 2020, these are the three visuals that have been most commonly associated with the virus and its implications. But a host of other visuals have also circulated widely in the news media at different times during the pandemic which have not garnered the same amount of attention or gained iconic status – the generic visuals that we describe above. It is these mundane visuals that have become the raw material for the everyday visual communication of the current global crisis. Given their ubiquity, understanding of the roles they play in representing something as significant and disruptive as a global crisis is needed. The coronavirus molecule image, the flatten-the-curve line graph, and the face mask visual are also standardized in significant ways, they are used as design elements in various layouts of news stories, and they circulate widely in the news media. However, unlike iconic visuals, we argue that generic visuals work through symbolic *reiteration* rather than symbolic *condensation*. The notion of symbolic reiteration is commonly used by literary and cultural studies scholars to examine how a ‘text’ – for example, a novel or a film – may reaffirm unspoken values through a variety of largely unremarkable rhetorical cues, to the extent that its ideological substrate may never become transparent even to the most attentive or informed reader.

While iconic visuals are by definition few and far between and therefore memorable as visuals in their own right, generic visuals are abundant and usually unremarkable as such. Generic visuals become meaningful through the repetition and recontextualization of their key semiotic resources and the symbolic patterns associated with them. In other words, generic visuals do not become recognizable as specific visuals (e.g. *that* photograph or *that* visualization). Rather, they mobilize visual concepts and motifs that become meaningful through their reiteration over time and across media outlets and media texts. The fact that the seemingly banal visual resources of generic visuals are repeated and repurposed across visuals is what interests us here. This is because this work of reiteration has important implications for how issues such as the coronavirus crisis are communicated to the public.

Our analysis foregrounds some of the ways in which generic visuals of Covid-19 in British news media promote routine engagement with a range of meanings which are affirmed by virtue of their reiteration. We illustrate this point below through a discussion of Covid visuals that we collected from Reach plc, BBC Yorkshire and the *FT* between December 2020 and January 2021, when the UK was in the midst of its ‘second wave’, and between April and May 2021, a less scary time as numbers of infections, hospitalizations and deaths had decreased considerably. This arc of events meant that the two time periods produced varied and rich visuals for us to examine. It also strengthens our argument regarding symbolic reiteration, in that we see the same types of visual resources and overarching themes emerge across a fairly diverse range of news stories and news media outlets. We collated our image sample for two weeks during the first period, and for a month during the second period. We limited ourselves to images that were online only, because online news is increasingly widely consumed and for ease of access. When encountering a story about Covid-19 on the front page of our selected publications, we selected the first image (for example stock photograph, cartoon, editorial photography) and all data visualizations. We gathered images in this way to ensure we collected both photos and visualizations. News stories tend to lead with a human interest visual, usually a photo, and data visualizations tend to appear later on in a story, so capturing only initial images would have skewed our sample towards photos. In total, there were 243 visuals in our sample, of which about two-thirds were photos and about a third were data visualizations, together with a handful of other types of visuals, such as infographics or composite images.

## Invoking banal belonging through symbolic reiteration in generic visuals of Covid-19

In this section we explore how, by virtue of symbolic reiteration, journalistic generic visuals of Covid-19 point publics towards assembling around a sense of ‘banal belonging’. We identified that key visual resources which are repeated across different visuals and repurposed to illustrate different news stories assemble audiences towards everyday forms of affiliation which are specifically national, and also sometimes local and global. Banal belonging may create different senses of possible public action, constructing Covid-19 as simultaneously global, national, and local (Anderson, 1983). Our concept of banal belonging derives from Michael Billig's research on banal nationalism, where he foregrounded the importance of ‘routinely familiar habits of language’ (Billig, 1995: 93) in political and news media discourse, as these ‘flag’ the homeland in ways that make it both ‘present and unnoticeable’ (Billig, 1995: 109). In this way, he argued, seemingly unremarkable linguistic patterns also powerfully point to nationhood. Together with everyday symbols, such as flags that are ‘unsaluted and unwaved’ (Billig, 1995: 40), commonplace words like ‘people’, ‘society’, ‘we’ and even ‘the’ (as in ‘the people’) contribute to reproducing the nation. Through the habitual repetition of such symbols and words, this work of flagging national belonging ‘is always a reminding, a re-presenting and, thus, a constricting of the imagination’ (Billig, 1995: 103). In other words, ‘banal’ nationalism is not meaningless or benign. Rather, it constructs and constrains the ways in which we view and inhabit national identity.

Existing scholarship on banal nationalism has mainly focused on language and, at times, on overt symbols of nationhood like, for example, flags, sports, or food (Skey, 2011). However, we see a similar dynamic at play in the news visuals of Covid-19 that we collected, in that generic visuals of the pandemic are often present and unnoticeable pointers towards nationalism. Although banal nationalism dominated, advancing Billig's concept, we also identified calls to banal localism and banal cosmopolitanism. These often occur separately, according to different news stories and news media outlets which themselves are usually local, national, or cosmopolitan in their focus and target audiences. However, at times generic visuals of Covid-19 assemble their audiences simultaneously to more than one form of belonging, in ways that reveal the complexity of news media discourse in the context of the pandemic crisis.

The intentions of the news professionals in creating these various kinds of banal belonging cannot be confirmed by an analysis of the images alone; these are best identified by talking to them. Likewise, to know whether audiences actually feel assembled to belong in the ways we suggest in our analysis, we need to ask them. These will be the next steps of our research into generic visuals in the news. For now, what this article does is to propose the concept of ‘symbolic reiteration’ as an analytical tool for making sense of generic visuals. At the same time, we advance the notion of ‘banal nationalism’ by pointing to the different kinds of banal belonging that generic visuals invite. We illustrate our overarching argument below, where we analyse key examples of banal nationalism, banal localism, banal cosmopolitanism, and of banal belonging across these ‘isms,’ in generic visuals in the news. Such analysis of generic visuals is important, we argue, because the subtlety of these different types of public assemblage would be lost through an analysis of only news texts, say, or of iconic images.

Generic visuals contribute to promoting specific visions and versions of belonging that become both pervasive and imperceptible through their volume and repetition of mundane visual resources and motifs. In particular, our sample revealed that, in addition to perhaps unsurprising markers of national identity, some visuals pointed to an imagined community assembled through the state, rather than through the everyday cultural markers that we have come to associate with ‘Britishness’. As Gerbaudo (2021) has recently argued, the global crisis brought about by the pandemic may have been a catalyst for a large-scale ideological shift from neoliberalism to ‘neo-statism’, or from widely held political beliefs in self-regulating markets and hands-off governments to growing cross-party demands for a more interventionist state. In our sample, state intervention can be seen in these generic visuals, often in subtle ways. At the same time, the question of ‘which state’ and ‘intervenes for whom’ also shadows these visuals, concepts we explore further below with our discussion of banal nationalism and banal belonging.

### Banal nationalism

**Figure 1.**
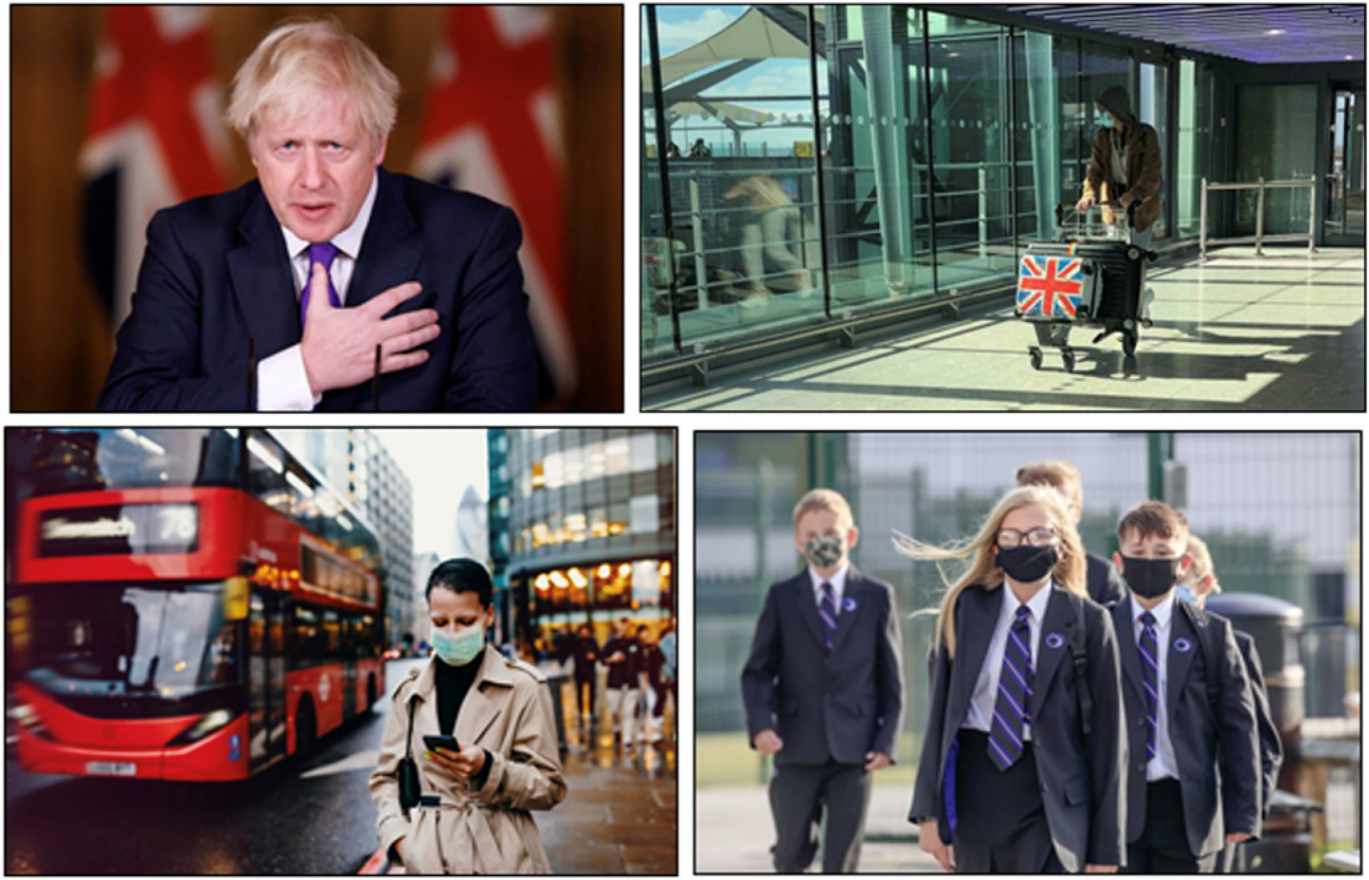
The Union Jack in the *Daily Star* (16 December 2020) and the *Financial Times* (3 January 2021, 6 April 2021) and representing Britain as ‘the context’ in a BBC news story (15 December 2020) and in the *Hull Daily Mail* (5 January 2021)

Our first set of findings pertains to generic visuals that promote an everyday affiliation with national identity. While the United Kingdom is a union rather than a nation, Billig (1995) himself points out that the Union Jack is often unobtrusively used to ‘mark’ belonging to the United Kingdom as a rhetorical nation. Photographs integrating the flag into their composition were common in our sample, for example in portraits of Boris Johnson in front of the flag or in stock photos where the Union Jack featured on the side of a suitcase (Figure 1). Many generic visuals of Covid-19 simply pointed to Britain, and more often than not specifically England, or what Billig (1995: 109) defined as ‘*the* context’, that is, the taken-for-granted ‘here’ of the pandemic. For example, stock images of people wearing face masks were often set against backdrops such as airports, schools, streets, which featured generically ‘British’ signage, settings and props such as school uniforms or modes of transport (Figure 1). ‘Britain’ was often indexed through visual resources that were specific to England, and London in particular. This visual ‘pointing’ to London as a metonymy for the UK was not always related to a news story focusing on London. A stock photo from Getty Images, where a double-decker bus (and arguably also the portrayed woman's trench coat) ‘stood’ for London, was used in a BBC article from December 2020 about harder ‘tier-three’ restrictions being introduced in London and South-East England. A similarly ‘London-centric’ stock image that portrayed a man standing on a train in the tube was used as an opening image in a BBC article about planned rules and guidance for face masks not only in England, but also in Scotland, Wales and Northern Ireland.

**Figure 2.**
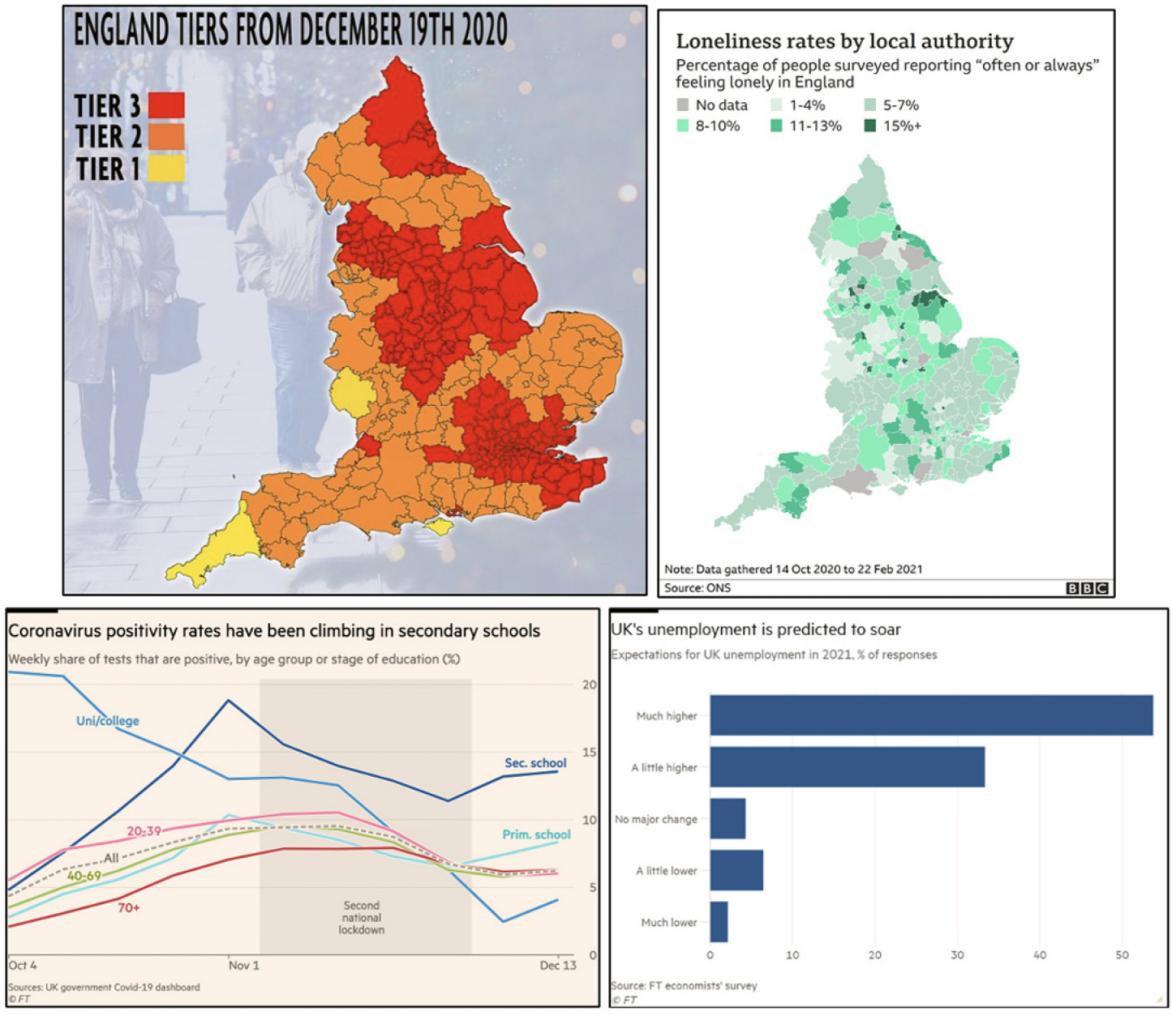
Visualizing England in maps from *The Mirror* (18 December 2020) and the BBC (3 May 2021) and the UK in line and bar charts from the *Financial Times* (17 December 2020, 3 January 2021)

Choropleth maps of England were mobilized regularly in BBC and Reach news stories on topics ranging from the introduction of new restrictions in the face of rising R rates to the relationship between lockdown and feelings of loneliness. In the same way, both the BBC and Reach also often produced charts of data broken down by counties or localities within the specific English regions that their publications represented (Figure 2). While the focus on England in these visuals may reflect the geographical reach of the related news media outlets, it is important to point out that stories featuring maps of England often also report on other parts of the UK in a fairly detailed manner. For example, *The Mirror*'s story from 18 December 2020 features two maps of England, while also reporting on R rates across the UK's four countries. In contrast, there were no maps of England or the UK from the *FT*, a broadsheet and more cosmopolitan publication, in our sample. This is despite the fact that most data visualizations in our sample came from the *FT*. Instead, the *FT* used bar charts and line charts to represent data which was sometimes about England, as in the line chart of coronavirus positivity rates in England from 17 December 2020. More typically, however, their data focused on the UK as a whole, as seen the bar chart about the predicted effects of Covid-19 on the UK economy (also in Figure 2). Like maps, the vast array of bar charts and line charts of Covid data – and, in the case of the *FT*, sometimes more complex graph types like scatterplots – also serve to suggest banal belonging to the nation, which is sometimes England and sometimes the UK.

**Figure 3.**
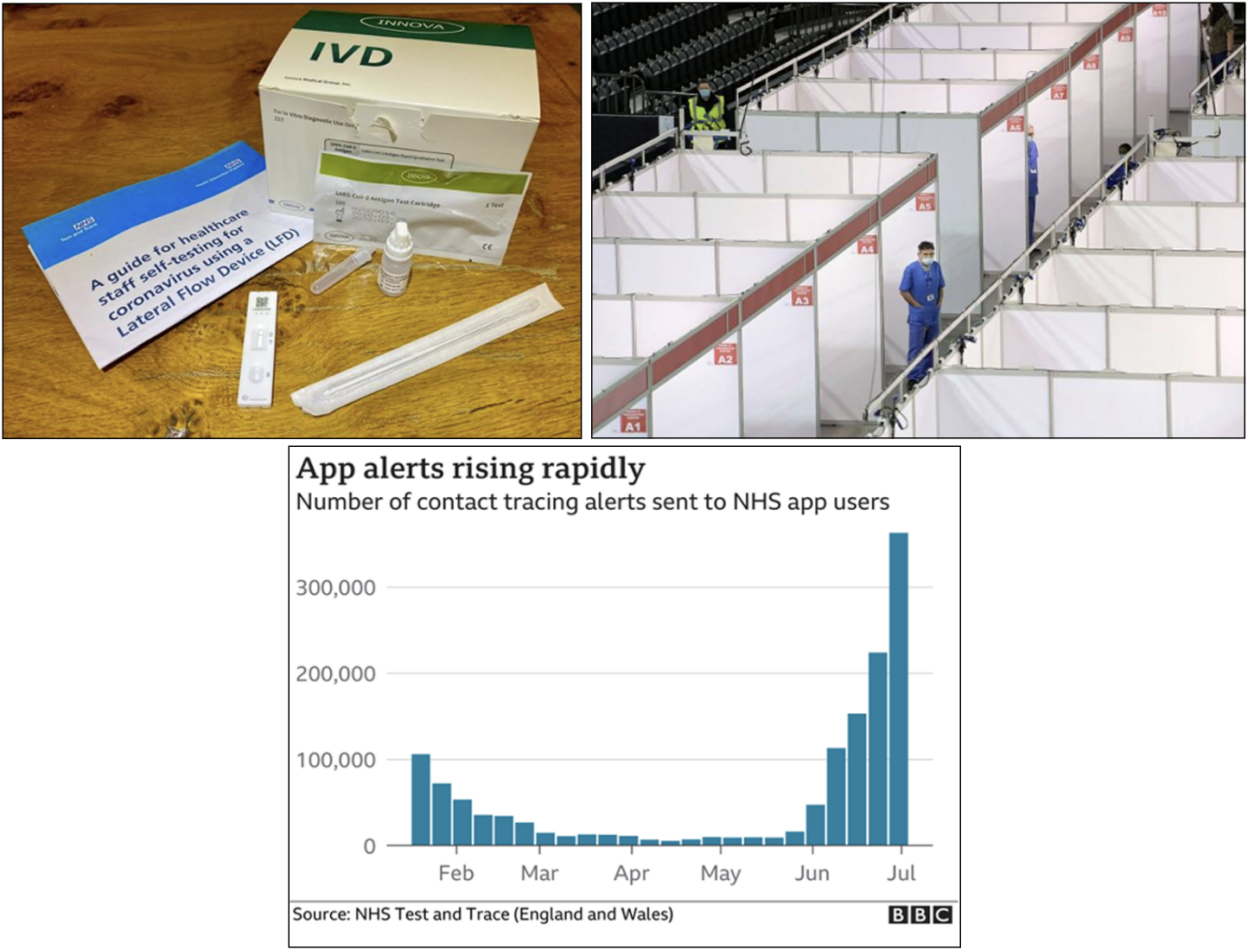
Generic visuals of the NHS from Teesside Live news website (14 December 2020), the *Financial Times* (7 April 2021) and the BBC (15 December 2020)

In contrast to this focus on England as a privileged national ‘backdrop’ for news of the various everyday implications of Covid-19, generic visuals of the National Health Service (or NHS) and of the UK's vaccination rollout – in which the NHS is a central actor – seemed to portray the UK as a fully united ‘national’ context. The NHS itself has been something of a rhetorical national flag during Covid-19, with its hard-working staff celebrated through weekly doorstep clapping during the early stages of the pandemic. This status is in part achieved thanks to the symbolic reiteration of the NHS’s role during the pandemic through generic visuals representing its infrastructure and interventions (Figure 3). While these visuals of state infrastructure and intervention may be less noticeable than visual resources like ‘British’ flags, props and maps, they are just as meaningful as markers of nationhood in the context of the Covid-19 crisis. Increasingly familiar and routine like the habits of language that Billig examines, we see stock photos of NHS testing kits and testing facilities as well as visualizations of NHS data across news media outlets. The NHS contact tracing app featured prominently in data visualizations and stock photos alike, for example in a BBC article on the NHS test-and-trace system from 15 December 2020 and a Bristol Live (news website) article about rules for visiting pubs and restaurants from 12 April 2021, respectively. Directly and indirectly, generic visuals pointing to NHS infrastructure and state interventions such as the deployment of soldiers to staff testing centres never highlighted regional or national differences, even in those cases where the caption revealed their specific location – such as in an example from the *FT*, where a photo of a rather generically pictured NHS testing facility had been taken in Belfast, Northern Ireland (Figure 3).

**Figure 4.**
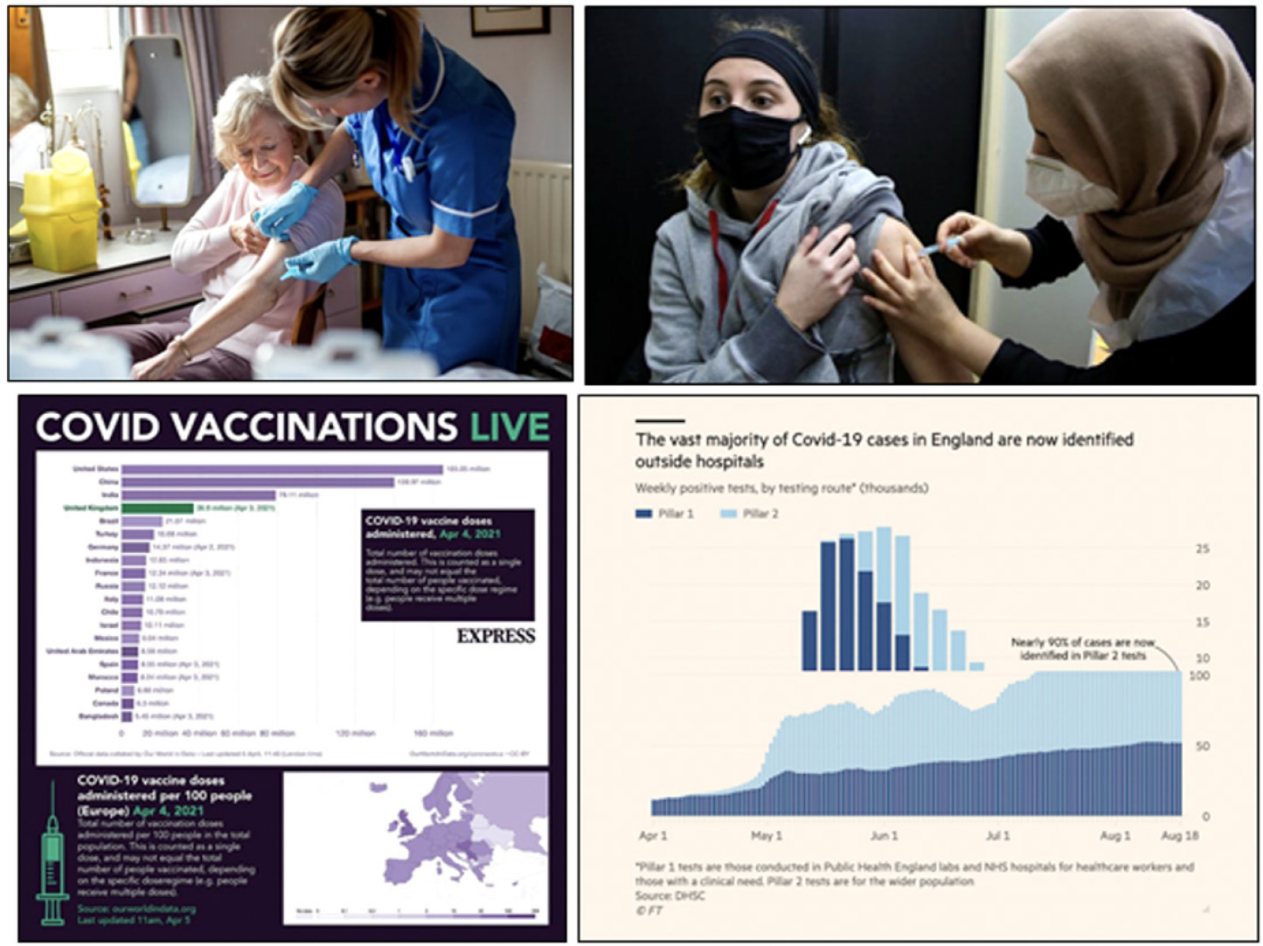
The NHS as a framework for national belonging: photos of vaccination in the UK from *The Mirror* (14 December 2020) and the *Financial Times* (7 April 2021) and visual representations of UK data about vaccination and Covid-19 cases from the *Express* (6 April 2021) and the *Financial Times* (1 December 2020)

Generic visuals of vaccination were also frequent. First announced in November 2020, the rollout of the various vaccines began around the world in early 2021 and by April had established itself as a mainstream news item of general interest. With the previous Covid wave and Brexit in the rear-view mirror, the success of the vaccine rollout in the UK became the focus of much pandemic media coverage. Here we found occasional visual representations that portrayed specific subjects in/or specific settings where vaccination occurred. For example, a Bristol Live article from 30 April 2021 used an image of Boris Johnson visiting the Ashton Gate vaccination centre as an opening visual, and similarly a *Mirror* article on the AstraZeneca vaccine from 7 April 2021 included a photo of Boris Johnson being vaccinated as its final visual. Overall, however, photographs of vaccination most often followed the same generic patterns, as they typically pictured female nurses giving patients their vaccination in their left arm while wearing blue medical-grade gloves and/or masks. At times, these were ‘proper’ stock photos, such as in the case of an image used to illustrate the section of a *Mirror* article from 14 December 2020 explaining that patients over the age of 80 would be vaccinated first. At the same time, we often also found similar photos that were captioned with the name of the vaccine recipient and the town or care home where the vaccination took place. The majority of these photos portrayed white people, whereas the few images that featured non-white people being vaccinated or vaccinating others were clearly marked as having been taken in areas with high levels of ethnic diversity (such as ‘North London’) or were specifically used to illustrate articles about vaccine uptake in Muslim communities (Figure 4). As a whole, images of vaccination painted a united though fairly homogeneous picture of the role of a ‘national’ institution, the NHS. The NHS stands in for the nation as a key framework for belonging together in the midst of the crisis.

The repeated representation of the NHS – through visuals of infrastructure, workers, and data – as a major framework for national belonging in the midst of a global crisis was also achieved through the myriad of simple data visualizations and other image types that have proliferated in news media during the pandemic crisis. The NHS is indirectly referenced in visual representations of vaccination rates and the impact of vaccinations on things like case rates in hospitals. Visual representations of data about the advantages and advances of the UK's vaccination programme, the overarching benefits of the UK's own vaccine, the success of the UK's approach to mass vaccination and visual representations of data about falling case rates in hospitals reiterate the role of the NHS in the fight against the pandemic without referring to it directly. In these visuals, UK data is not, on the whole, broken down by country and, as result, it is the union, rather than any one of the union's four countries, to which we are invited to feel a sense of national belonging (Figure 4).

**Figure 5.**
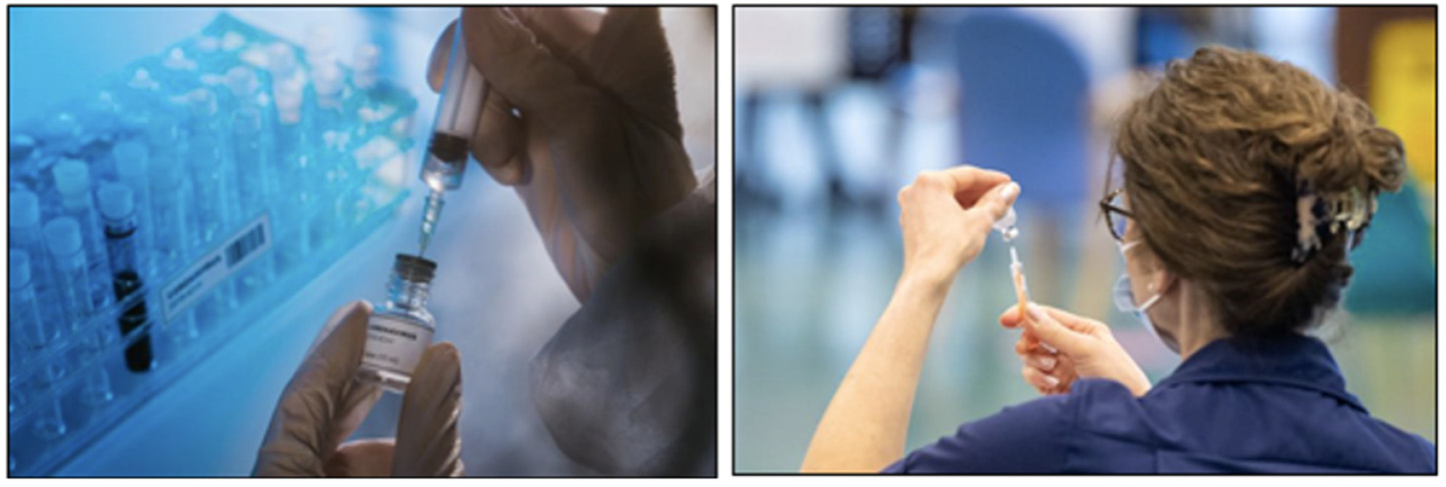
Stock photos of vaccine preparation from *The Mirror* (14 December 2020) and Hull Live news website (17 April 2021)

The symbolic reiteration of the role of the state via vaccination and healthcare also occurs through the deployment of stock images where the act of preparing or administering vaccines is decontextualized from settings like GP’s surgeries, care homes, pharmacies, or community centres, and where we do not see those who are about to get vaccinated and the faces of the healthcare workers preparing the vaccines are often blurred or excluded (Figure 5). These more abstract images still work performatively to promote banal nationalism as mediated by state intervention and infrastructure, insofar as they foreground the act of preparing a vaccine and the implication that it will be soon administered to an ordinary citizen as central to the ‘story’. And precisely because of their more conceptual rather than descriptive or editorial nature, these are also arguably images that can be and are used by a variety of national and international news media outlets to invite their audiences to feel a sense of belonging to a national collectivity supported by the state. Although the banal visuals of Covid-19 in our sample most frequently assembled a national public, some generic visuals also spoke to a sense of banal localism and some to banal cosmopolitanism. A small amount of images invited banal belonging in ways that referenced two or more of nation, locale and world. We discuss some examples of these images in the next section.

### Banal localism, banal cosmopolitanism, and banal belonging across ‘-isms’

**Figure 6.**
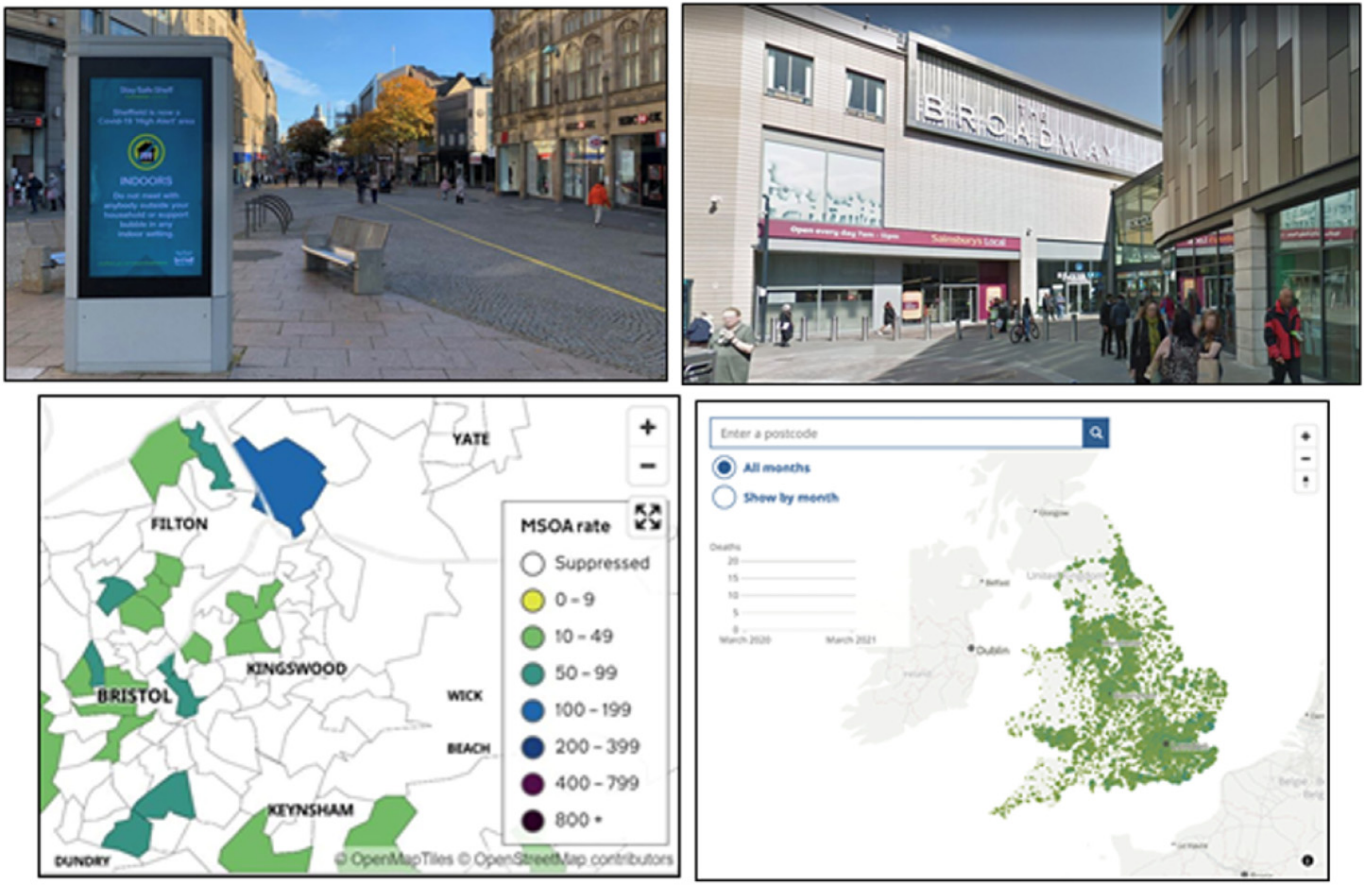
Banal localism in generic photos of local landmarks such as the ‘high street’ in Sheffield, South Yorkshire (BBC, 12 December 2020) and the Broadway shopping centre in Bradford, West Yorkshire (BBC, 12 April 2021) and in maps showing Covid-19 data in Bristol Live (3 May 2021, 30 April 2021)

With regard to generic visuals invoking banal localism, a first key example pertains to the routine presence of generic images of local landmarks, particularly in news stories from BBC Yorkshire and newspapers from the Reach plc group. Unsurprisingly, the news stories where we found these photos were indeed about local matters like, for example, the opening of new vaccination hubs in Leeds or an outbreak among staff members at an Exeter Sainsbury's. And while photos of locales like the Elland Road Stadium in Leeds or the Sainsbury's on Pinhoe Road in Exeter were appropriately used to illustrate stories like the ones we have just described, these visuals are also ‘generically local’. This is both because they portray specific locales, for example the Broadway shopping centre in Bradford as seen in Figure 6, through medium to wide shots and neutral angles that present them as typical of their kind (Kress and Van Leeuwen, 2021), but also because they visually point to features of the built environment that are translocally mundane. For example, in the shot of a Sheffield city centre view, also seen in Figure 6, used to illustrate a 12 December 2020 BBC news story on the likelihood of tier-three restrictions for South Yorkshire over Christmas, on the left we see signage stating key guidance on indoor mixing while in lockdown, whereas the remaining two-thirds of the image, including the centre, portray a ‘high street’ like many others in the UK. Along the same lines, the stadium, the shopping centre and the supermarket are very much part of everyday experiences of living in a particular city or town, and as such these stock images of mundane landmarks invoke an affinity with the local. Not surprisingly, regional publications in the Reach plc group and the BBC Yorkshire website also often showed maps of local areas, which included data about a range of Covid-19 related issues. Examples of data represented include area-by-area infection rates within regions, data about neighbourhoods with the highest case rates in a region, or data predicting the lockdown status of different areas. These generic visuals invite a more localized sense of belonging, symbolically reiterating the geographic contours of an area or region, contours which themselves are most likely to be familiar to the people who reside therein. Sometimes maps of England and Wales also invite a sense of local belonging. The final image in Figure 6 shows an interactive map from Bristol Live on 30 April 2021 of death rates in local areas. Readers are invited to input their postcodes, and the map zooms into a very localized area to display the relevant information. Thus, the visual trope of the national map can invite a local, rather than national sense of belonging.

**Figure 7.**
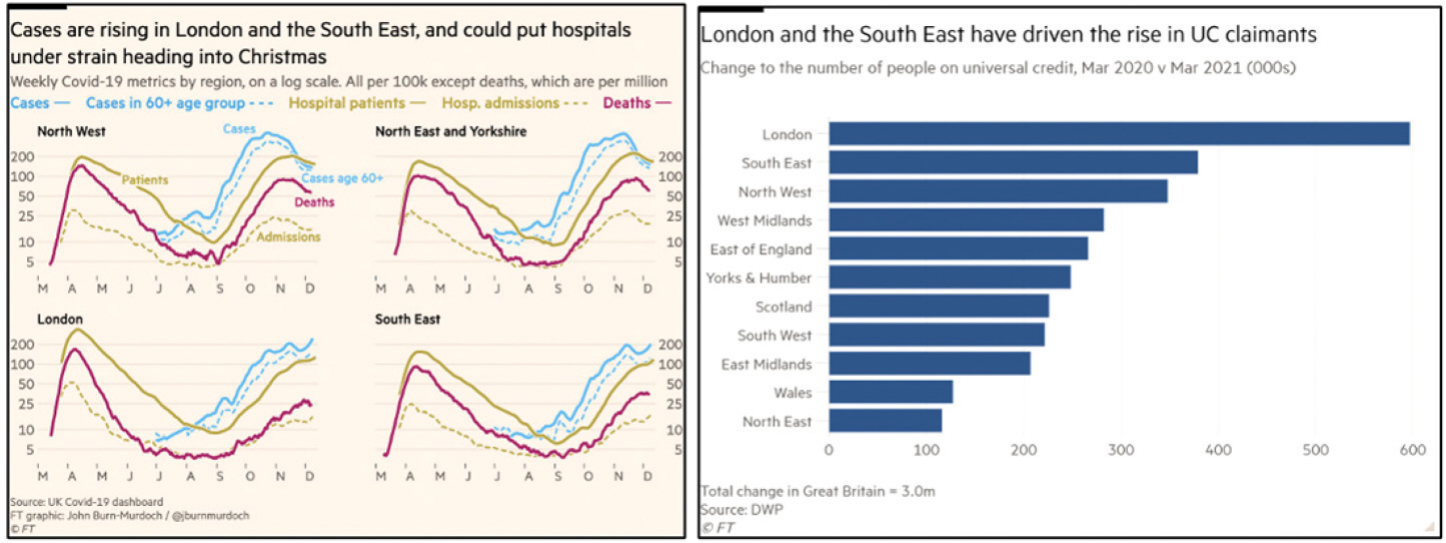
London-centric banal localism in simple data visualizations from the *Financial Times* (15 December 2020, 5 May 2021)

Although the *FT* is a more international publication than the others in our sample, it also sometimes includes examples of banal localism. In Figure 7, one example shows a line chart of rising Covid case numbers in London and the South East from 15 December 2020, the time when what would come to be known as ‘the Kent variant’ was emerging. An article from 5 May 2021 includes three visualizations which centre on the effects of Covid-19 on London: a bar chart comparing numbers of welfare benefit claimants in London with other regions, another bar chart of financial redistribution across regions, and a line chart comparing footfall recovery across London areas. It seems that, for the *FT*, local means ‘London’, an understanding that reflects the newspaper's focus on international financial affairs, at the centre of which the city sits.

**Figure 8.**
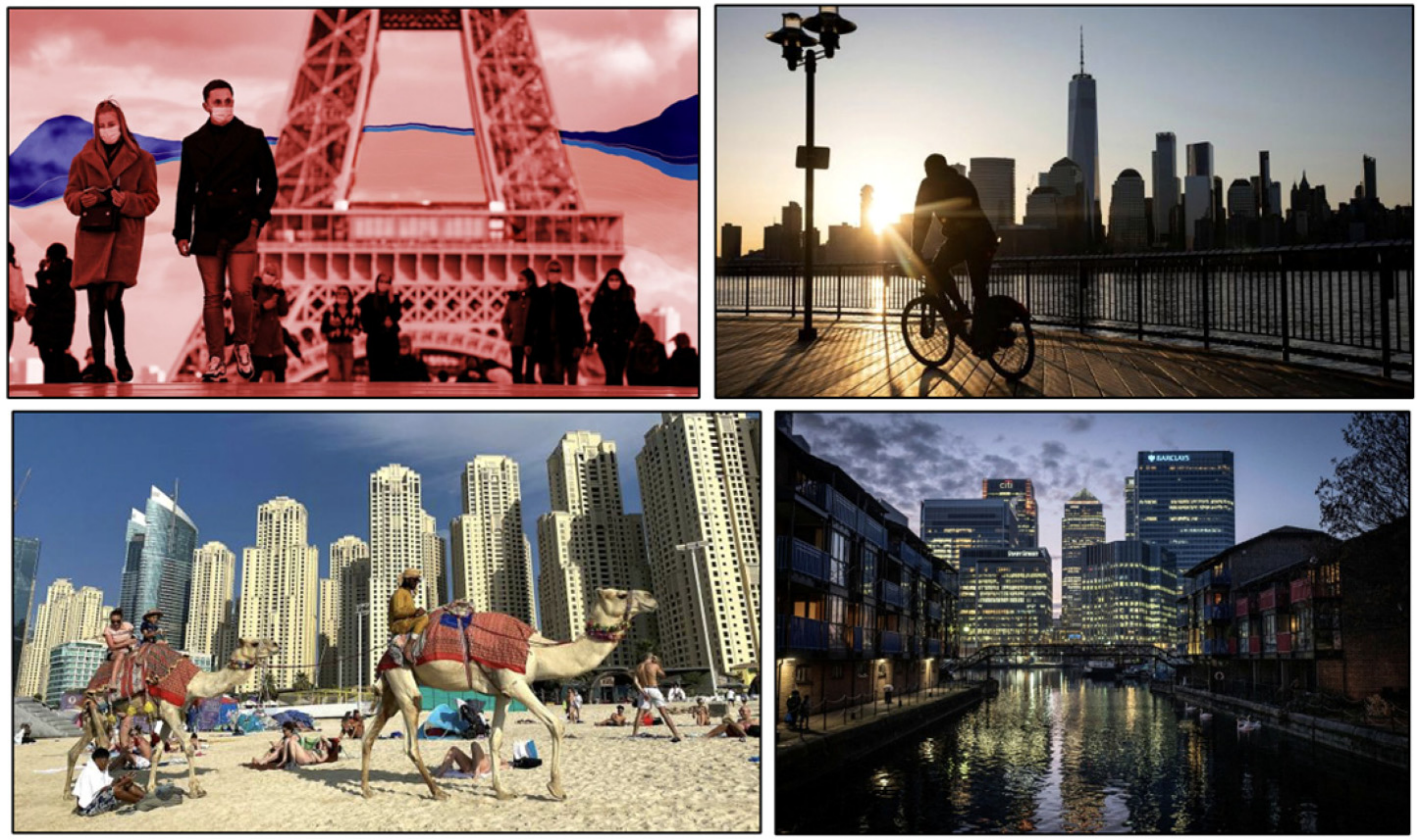
Banal cosmopolitanism through stock photos of cityscapes in the *Financial Times* (1 December 2020, 6 April 2021, 9 April 2021, 5 May 2021)

Much more common in the *FT* is a more cosmopolitan story, told through photos of the skylines of global cities and of global Covid data*.* Stock photos of internationally known cityscapes such as those of Paris, New York, Dubai and, of course, London (Figure 8) are mobilized in news stories on topics ranging from pandemic-related damage to advanced economies and the human cost of Covid-19 globally, to restrictions to international travel from the UK and the development of a vision for the UK capital's recovery. Overall, then, these images have a fairly tenuous connection to the content of the news stories that they accompany; however, they are mobilized as visual shorthand of a global urban ‘whole’, thus also assembling *FT* readers as cosmopolitan subjects who may feel at home in any one of these cities. While people are sometimes present in these stock photos, the human geography of the global cityscapes that they depict is not the main focus. Rather, these photos centre skylines and the built environment as shot from the distance, resulting in ‘disembodied and detached' (Aiello and Thurlow, 2006: 152–153) views that privilege the mundane materiality of these cities’ physical geography over their particularities or idiosyncrasies, and in this way also invoke banal belonging.

**Figure 9.**
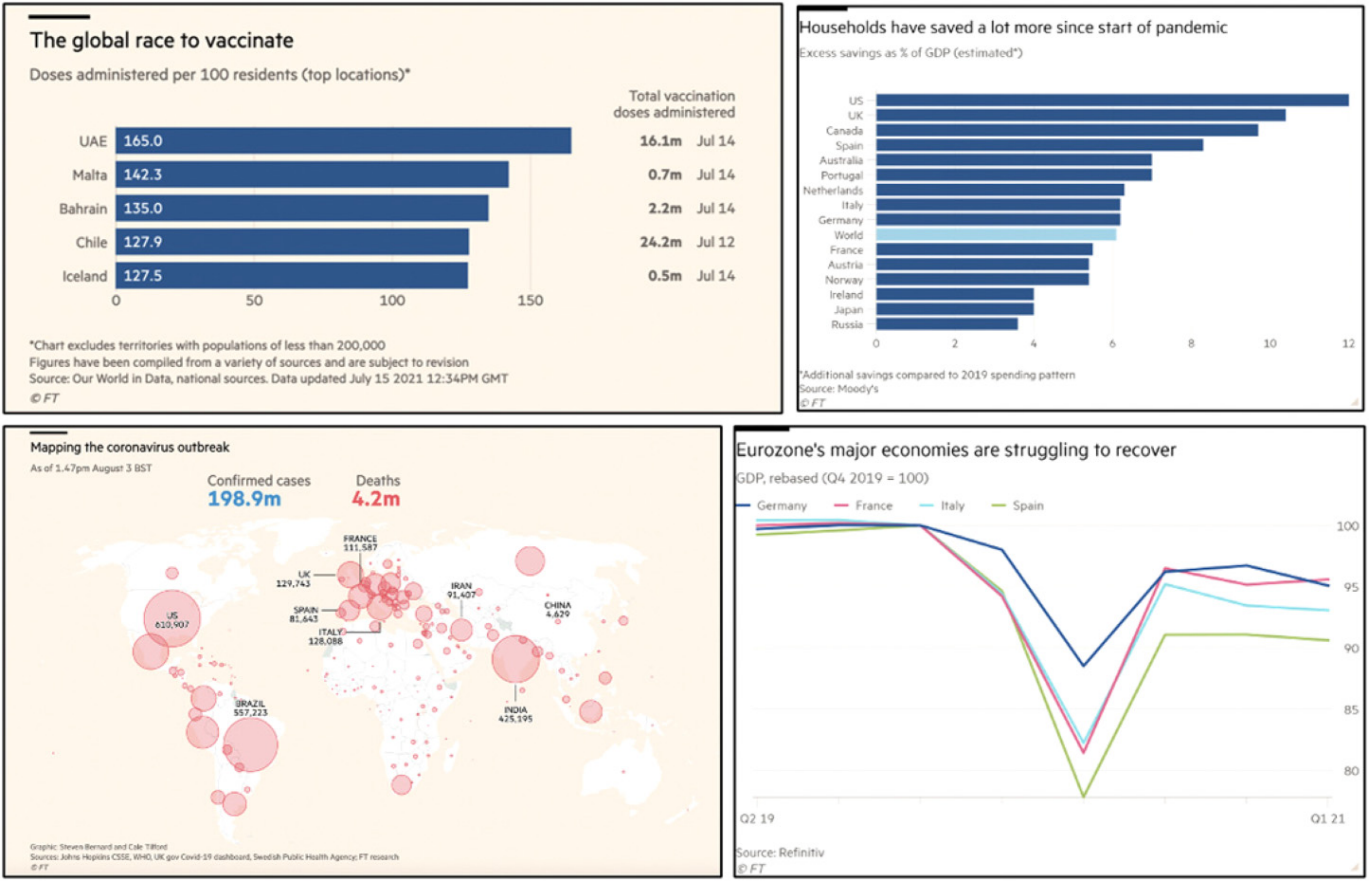
Banal cosmopolitanism in simple data visualizations from the *Financial Times* (1 December 2020, 16 April 2021, 21 April 2021, 30 April 2021)

Visualizations invoking banal cosmopolitanism include maps of different parts of the globe showing case rates, line charts of death rates across countries, some of which follow a similar shape to the iconic flatten-the-curve graphic, bar charts comparing a range of Covid-related matters, from grants for European Union (EU) member states to counter Covid-19 recession and the global race to vaccinate, to how household savings during the pandemic vary across country, and line charts relating to speed of economic recovery in different parts of the globe (Figure 9). Visualizations which reflect banal cosmopolitanism by comparing pandemic phenomena across countries also create a sense of national belonging to some extent, by situating what's happening in the UK in the context of other countries. Thus, just as the localism in the *FT* speaks also to the cosmopolitan, so too the cosmopolitan speaks to the national.

Particular pages in the publications we examined also invite their audiences to feel more than one form of belonging through simple data visualizations. These are often ‘tracker’ type pages, collating the latest data about Covid-19. The *FT*'s coronavirus tracker is one example.^1^ This dynamic page was updated across the two time periods of our sample with different kinds of data, sometimes global, sometimes about the UK. For example, in the first period, maps of different parts of the world and line charts of rising and falling cases sat alongside visualizations of where most Covid cases could be found in the UK. In the second period, there were more global comparisons – the same rising and falling line charts of case rates, a bar chart comparing excess deaths around the world, and line charts showing differences in hospitalizations, ICU (intensive care unit) patient numbers and deaths by age, in different parts of the world, from Chile to the US. Similar data was also provided, though in separate line charts, about the UK, alongside a line chart showing how vaccination reduces case rates by age group. These comparative trackers quite often resembled a (very deadly) global sporting event, in which nations were presented as if in competition with other countries to do a better job of managing the global pandemic.

It ought not to surprise us that whether the news media outlets we examined prioritize local, national or cosmopolitan news stories and accompanying generic visuals relates to the audiences they serve – some are regional publications, some are national, and some, like the *FT*, have a simultaneously national and international readership. But it is not simply the case that generic visuals in regional publications map onto a kind of banal localism, nor that national media outlets always talk about national issues. In part this has to do with the complex structure of ‘federal’ news organizations like the BBC, where national news stories and accompanying generic visuals can appear on the landing page of a regional website. But in part, it also has to do with the larger idea of the nation, assembled as a public through the news media, and the way that nation can serve as an object of intervention on behalf of the state. What our analysis has highlighted here is that ideas of belonging, grounded in a multitude of subtle ways through the use of generic visuals and symbolic reiteration, are as much about inclusion and exclusion as they are about nationalism or localism per se.

## Conclusion: generic visuals of Covid-19, banal belonging and the role of the state

Before we turn to our conclusions on the findings that we have just examined, we would like to reflect on whether bringing stock photos and data visualizations together as we have done here is productive or is not. We conclude that it is, even though the kind of belonging that they invite is somewhat distinctive for each image type. Stock photos invite a sense of banal and primarily national belonging by depicting people and places within which audiences may see themselves. Visualizations may appear to present more abstract and less familiar phenomena to which it is harder to relate – namely, data – and yet, as one of us has noted elsewhere, emotions are a vital component of making sense of data, especially when it is represented visually (Kennedy and Hill, 2018). This ‘feeling of numbers’ may then invite audiences to feel a sense of banal belonging, just as photographic images do. What's more, examined alone, simple data visualizations may appear only to inform, but examined together with other generic visuals, a lens of symbolic reiteration reveals the work that they do in assembling publics.

We have shown many stock photos and simple data visualizations relating to Covid-19 in this article, because a multitude of such visuals circulate every day in the news media. Like more iconic visuals of the pandemic, generic visuals are often standardized in form, they serve particular design functions, and they are increasingly ubiquitous. Unlike iconic visuals, however, generic visuals of Covid-19 are not memorable and immediately recognizable as visuals in their own right. Generic visuals do not demand attention and invite recollection. They are not ‘symbolic condensations’ channelling culturally meaningful issues and historically significant events into one or few particular aesthetic forms. Rather, generic visuals work through symbolic reiteration, reaffirming unspoken claims and even burgeoning values through a variety of largely unremarkable rhetorical cues – flags, maps, vaccine vials, bar charts, healthcare staff at work, everyday life locales and activities, rising and falling lines.

These ‘routinely familiar habits of [the] language’ (Billig, 1995: 93) of generic visuals of Covid-19, we argue, invite news media audiences to feel a sense of banal belonging, primarily to the nation, or the union in the case of the UK. Flags, public transport, school uniforms, data visualizations of Covid cases, deaths, vaccinations, and economic effects point to Britain, or more specifically England, as ‘*the* context’ (Billig, 1995: 109), the ‘here’ that matters in the pandemic. Some generic visuals invite banal belonging to the local area, and some also construct what we describe as banal cosmopolitanism, but the nation remains present in many of these, such as the *FT*'s focus on the UK capital, London, a centre of national and international financial affairs. In comparisons of global Covid-19 data the nation is still centred, inviting audiences to check how UK cases, deaths and vaccine rates compare with those of other nations. And through this centring of the nation, we also see the foregrounding of the state as a channel for national belonging in the face of the crisis. The state surfaces as important in this way because, for better or worse, the past two years have seen the largest state intervention into the lives of ordinary citizens that most of us in the post-Communist West have ever experienced. What's more, this intervention has been primarily nationalist in nature, with very little cosmopolitan cooperation. Borders suddenly re-emerged for EU citizens within the Schengen travel area, as countries fought to keep the virus at bay. In *Seeing Like a State*, James C. Scott (1998) argues that states render society legible. Following the lead of science and technology scholars we could turn this around to argue that nations must exist in order to be operated upon by states (cf. Law, 2008). In other words, assembling audiences around notions of banal belonging fosters a sense of nationhood which frames Covid-19 as a problem whose solution lies with the state (rather than at the global level). Our analysis reveals the ambient visual background within which these interventions took place. The forms of banal belonging represented in the generic visuals that populate the pages of the daily news reinforce state power, however unacknowledged and unnoticed. For this reason alone, these often-overlooked visuals are objects worthy of study.

Another thing that ought not to surprise us is that complex issues like managing a global health pandemic get reduced to simple messages about the importance of the nation and suggestions that the state is doing well in responding to the crisis. However, while we may expect the news media in general to simplify, to produce an ‘us’ (= the nation) vs. ‘them’ (= the rest) dichotomy, until now we did not know how generic visuals contributed to this agenda. Our overarching research aim is to explore whether generic visuals assemble publics, and from our analysis here, they appear to have the ability to do so. They contribute to a sense of banal belonging, assembling publics through the framework of state intervention. And while, according to Gerbaudo (2021), state interventionism will soon become the ‘new normal’ across the political spectrum, our analysis of generic visuals of Covid-19 has enabled us to examine some of the cultural mechanisms through which this process is taking place. It has also enabled us to trace some of the novel ways in which banal belonging, and banal nationalism in particular, is imagined and communicated in everyday media culture in the wake of the Covid-19 crisis and beyond.

## Notes

1. See: https://www.ft.com/content/a2901ce8-5eb7-4633-b89c-cbdf5b386938
